# Report of Diseases

**Published:** 1819-07

**Authors:** 


### REPORT OF DISEASES.

OUR various sources for information have furnished us with but little that has been remarkable in the diseases of the preceding month. No malady appears to be so especially prevalent as to be particularly designated, according to the views with which this Report is made. The fever which has often formed the subject of our observations, still exists; but, from the want of any extensive establishment appropriated for it, it is difficult to ascertain, with much accuracy, to what extent: we believe that this is not considerable, and that it is on the decline. The lungs and brain are now the organs chiefly implicated in the local disease with which it is accompanied.

Scarlatina of a mild character, and without much affection of the throat, is of rather frequent occurrence, and usually terminates in the most favourable manner, under a cooling regimen and restricted diet.

We have seen a few cases of dropsy succeeding to intermittent fever, in the lower class of the applicants to the public dispensaries. These have been successfully treated, speaking generally, by laxatives and digitalis. In one recent case, the fever (tertian) returned after the use of some violent purgatives taken by the patient, a middle-aged mother, without medical advice. The dropsy is now disappearing, and the state of the system in the intervals of the febrile paroxysms is more nearly approaching in appearance to that of health. Leeches to the abdomen, the warm bath, and a spare diet, have been the remedies. It is proposed to employ cinchona in a few days.

Several very troublesome cases of dyspepsia have appeared in elderly persons, who were ordinarily disposed to that affection, apparently solely in consequence of a free use of purgatives, (in general, salts, alone or combined with senna, but sometimes some of the resinous extracts,) which they took from a notion that they would be salutary at the present season. After all the ordinary remedies had failed to give relief, pills composed of rhubarb and ginger, (containing from five to ten grains of the former,) taken daily, so as to cause a little constipation of the bowels, have produced the most prompt and decisive good effects. One patient, a man of a spare habit, aged about 65 years, is invariably affected with flatulence, spasms of the stomach, vomiting after taking food, and acidity in the stomach, whenever his bowels are lax; and is immediately restored to health by the rhubarb-pills, which induce constipation.

